# The odor of a plant metabolite affects life history traits in dietary restricted adult olive flies

**DOI:** 10.1038/srep28540

**Published:** 2016-06-24

**Authors:** Christos D. Gerofotis, Charalampos S. Ioannou, Christos T. Nakas, Nikos T. Papadopoulos

**Affiliations:** 1 Laboratory of Entomology and Agricultural Zoology, Department of Agriculture Crop Production and Rural Environment, University of Thessaly, Phytokou St., 38446 N. Ionia Magnisia, Greece; 2 Laboratory of Biometry, Department of Agriculture Crop Production and Rural Environment, University of Thessaly, Phytokou St., 38446 N. Ionia Magnisia, Greece; 3 University Institute of Clinical Chemistry, Centre of Laboratory Medicine, Inselspital, Bern University Hospital, University of Bern, 3010 Bern, Switzerland

## Abstract

Food quality shapes life history traits either directly or through response of individuals to additional environmental factors, such as chemical cues. Plant extracts used as food additives modulate key life history traits; however little is known regarding such effects for olfactory chemical cues. Exploiting an interesting experimental system that involves the olive fly (*Bactrocera oleae*) and the plant metabolite α-pinene we asked whether exposure of adults to this compound modulates adult longevity and female reproduction in similar manner in a stressful – dietary (protein) restricted (DR) and in a relaxed- full diet (FD) feeding environment. Accordingly, we exposed males and females to the aroma of α-pinene and measured lifespan and age-specific fecundity in the above two dietary contexts. Our results demonstrate that exposure to α-pinene increased longevity in males and fecundity in females only under dietary restricted conditions. In relaxed food conditions, females exposed to α-pinene shifted high egg-laying towards younger ages compared to non-exposed ones. This is the first report demonstrating that a plant compound affects key life history traits of adult olive flies through olfaction. These effects are sex-specific and more pronounced in dietary restricted adults. Possible underlying mechanisms and the ecological significance are discussed.

Among the various challenges organisms face towards surviving and reproducing, quantitative and qualitative limitation of food stands out. Adaptive and/or plastic competence of living creatures to cope with adverse feeding conditions, such as scarce availability and low nutritional value of food, determines thriving and existence. Perhaps one of the most dramatic cases of environmental variance is living in high versus low nutritious environments (relaxed vs stressful respectively). Nutrient acquisition can generate variation in the amount of resources available for the individuals and may moderate the severity of trade-offs between survival and reproduction^1^,^2^. Caloric and/or dietary restriction may modulate the response of an organism to environmental stimuli^3^ eliciting hormetic responses in a variety of animal taxa^4^. Food quality, therefore, is expected to shape (positively, moderately or negatively) life history traits^5^ through successful response of individuals to additional environmental factors, such as chemical cues.

Plant extracts evoke various responses in several animal taxa (humans, insects, worms, etc.) modulating key life-history traits, prolonging lifespan and enhancing stress tolerance^6^, increasing fecundity^7^ and affecting behavior and physiology of organisms (for reviews see^8^,^9^). Plant compounds that are incorporated in food as diet supplements prolong longevity in model organisms by interacting either with nutrient-, energy- and stress-sensing pathways^9^ (and references therein). Among the many compounds tested, resveratrol constitutes probably the best studied phytochemical that prolongs longevity^10^,^11^ (and references therein). Likewise, blueberry extract extends the lifespan and increases thermotolerance in the nematode *Caenorhabditis elegans*^12^. Nectarine extracts have been shown to extend lifespan and simultaneously increase fecundity in *Drosophila melanogaster*^7^. Mixture of oregano and cranberry has been demonstrated to increase longevity without effects on the fecundity of Mexican fruit flies (*Anastrepha ludens*)^13^. Extracts of the acai berry improve survival of flies feeding on a high fat diet^14^. Despite being used as food additives the underlying mechanisms of extending longevity and modulating life history traits differ a great deal among the above compounds ranging from anti-oxidant, anti-flammatory, to immune-stimulatory properties. There is a great variation in the outcome of the above studies demonstrating positive effects of plant extracts used as food additives, which depend on the gender of tested individuals, diet conditions and other environmental factors.

Olfactory chemical cues -including phytochemicals- emitted from host plants of phytophagous insects may elicit a wide range of behavioral and physiological responses^15^, providing important signals for locating food sources^16^, oviposition sites and/or mediating oviposition behavior^17^,^18^. Phytochemicals may generate similar reactions in biochemical and physiological pathways towards increasing longevity as those reported earlier for plant extracts used as food additives, though through neurosensory regulation. Sensory cues (food and pheromone odors as well as additional environmental cues such as temperature) have been found to modulate longevity and reproduction in model organisms^19^,^20^,^21^,^22^, (reviewed in^23^). Food and carbon dioxide volatiles have been shown to expand longevity in *Drosophila melanogaster*^20^,^24^. Perception of olfactory, gustatory and thermosensory stimuli can modulate lifespan in *C. elegans* as well^22^,^25^,^26^. While there is a plethora of studies depicting beneficiaries of phytochemicals when added in food, very few refer to positive effects through olfactory perception.

The olive fly, *Bactrocera oleae* (Rossi) (Diptera: Tephritidae) is a rather monophagous species feeding on cultivated and wild growing olives. Similar to other true fruit flies (tephritids) females lay eggs in olive fruit and larvae feed in olive fruit flesh before pupating^27^. Alpha-pinene, a common plant compound that is present in both olive fruit and leafs, consists also one of the four major compounds of the sex pheromone of female olive flies^28^ that has been found to evoke physiological responses and modulate behavioral ones in both male and female olive flies. A-pinene exhibits synergistic action with olean (the main compound of the female pheromone blend) in attracting males^29^ and in stimulating oviposition^30^ and most probably is acquired by olive leaves and/or olive fruit. More recent studies demonstrate that exposure to the aroma of α-pinene evokes strong electrophysiological responses in both male and female antennae^31^, and increases the mating performance of both sexes^32^. Thus, there is an intimate link between olive fruit flies and α-pinene since both adults and immatures (developing in α-pinene rich olive fruit) are frequently exposed to this compound. Similar to other fruit flies protein limitation in the adult food of the olive fly is expected to establish a nutritionally stressful environment^33^,^34^,^35^. Working towards exploring how chemical cues- closely related to the ecology of a species- can influence longevity and reproductive life history traits of olive flies and whether these effects are modulated by the dietary conditions that adults experience, we asked the following questions: Does exposure to α-pinene modulate: (a) longevity in olive flies, (b) reproductive traits of females and (c) life-history traits in similar manner in a dietary restricted (DR) and in a relaxed- full diet (FD) feeding environment. Accordingly by manipulating the availability of yeast hydrolyzate in adult food and exposure to α-pinene we tested the hypothesis that exposure to α-pinene influences longevity and reproduction regardless of dietary restriction, and the gender of individuals.

## Results

### Effect of α-pinene on adult longevity

**Figure 1:**
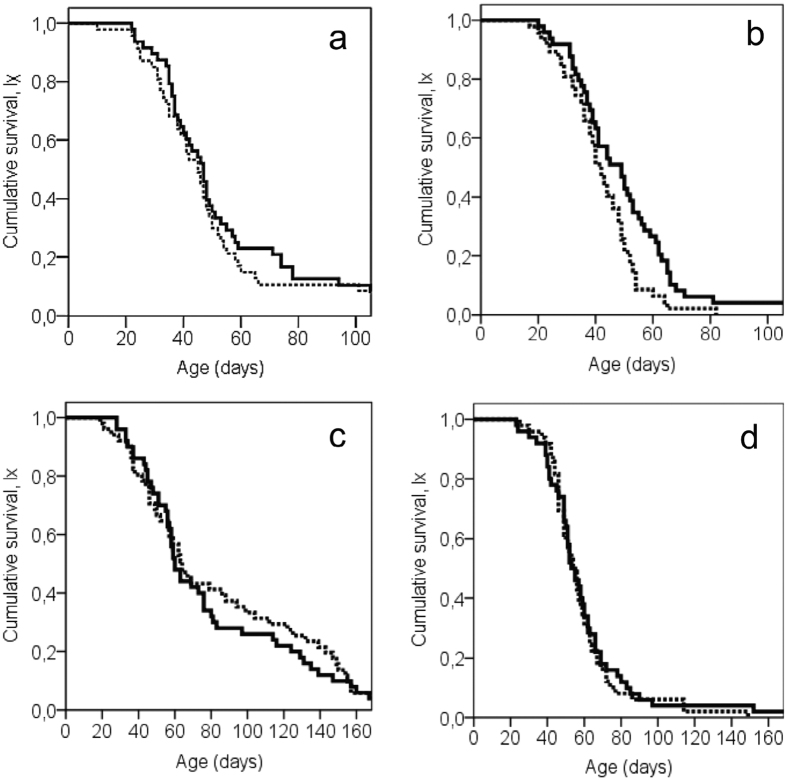
Age-specific survival patterns of adult olive flies that were either exposed (solid line) or non-exposed (dashed line) to α-pinene: (**a**) males in full diet (FD) conditions, (**b**) males in diet restriction (DR), (**c**) females in full diet (FD) conditions, and (**d**) females in diet restriction (DR) conditions. (Log-rank pairwise tests reveal significant differences between exposed and non-exposed males fed in sugar, *P* < 0.05).

Table 1 gives the longevity parameters of both male and female cohorts and Fig. 1 the respective cumulative survivorship curves. Exposure to the aroma of α-pinene significantly increased the lifespan of DR males only (log-rank test; *x*^2^ = 6.027; df = 1; *P* = 0.014). There was a positive response to α-pinene in terms of longevity extension of males’ experienced FD conditions but the difference between exposed and non-exposed individuals was not significant (log-rank test; *x*^2^ = 0.417; df = 1; *P* = 0.518). Exposure to α-pinene did not prolong the longevity of females regardless of the food regime (Table 1; Fig. 1c,d, log-rank test; *x*^2^ = 0.403 and *x*^2^ = 0.197; df = 1; *P* = 0.525 and *P* = 0.657).

### Effect of α-pinene on female reproductive traits

**Figure 2:**
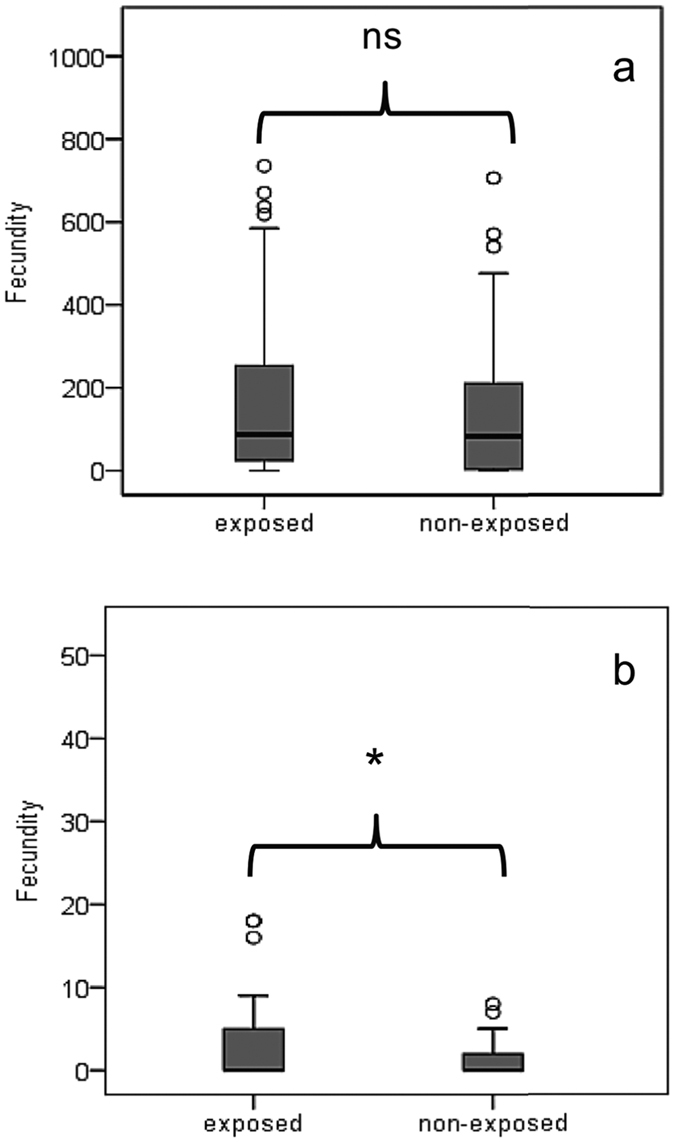
Box plots depicting life time fecundity rates (eggs per female) of females experiencing FD (**a**) and DR (**b**) food conditions and had either exposed or remained unexposed to α-pinene. Asterisk indicates significant differences between female cohorts (squared Wald test *x*^2^ = 81.219; df = 1; *P* < 0.05).

**Figure 3:**
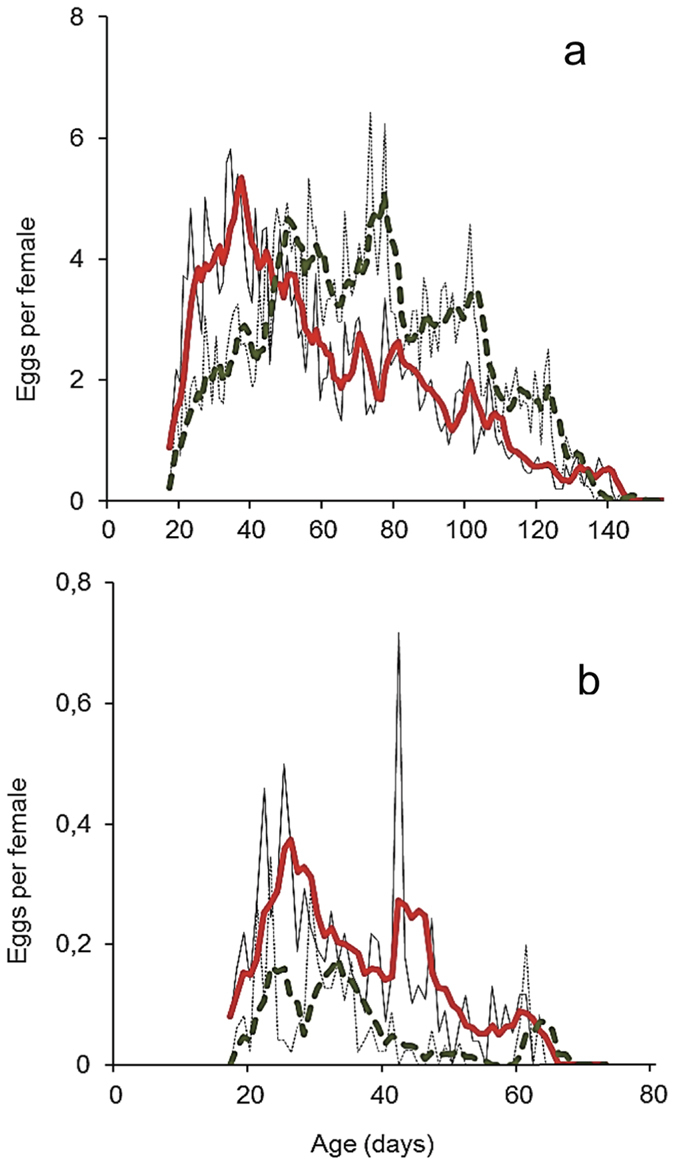
Age-specific fecundity of exposed (solid black line) and non-exposed (dashed black line) females in FD (**a**) and in DR food conditions (**b**). Red solid (exposed) and green dashed (non-exposed) lines represent data of female fecundity after smoothing of 5-days period, in the respective food regimes.

Figures 2 and 3 give the average lifetime and age-specific fecundity rates of females’ experienced FD (a) and DR (b) nutritious conditions respectively. Within cohort variability in age specific egg-laying patterns is given in Supplementary Figure 1. Exposure to α-pinene increased the number of eggs oviposited by females experiencing DR conditions (squared Wald test, *x*^2^ = 81.22; df = 1; *P* < 0.001, Fig. 2b), but not that of females having access to FD environment (*P* = 0.426). Interestingly, exposure to α-pinene shifts intensity of egg-laying to younger ages for females feeding in FD but not for those feeding in DR food (Fig. 3). ROC curve analysis (Supplementary Figure 3) highlights differences in the age-specific distribution of the egg laying output between non-exposed and exposed to α-pinene females that experienced FD environment (Supplementary Figure 3a, *P* = 0.002). The distribution of the egg laying output was similar between exposed and non-exposed to α–pinene females in the DR environment (Supplementary Figure 3b, *P* = 0.62). Exposure to α-pinene substantially (from 32.7 to 48.0%) and slightly (82.4 to 88.0%) increased the proportion of ovipositing females in cohorts maintained in DR (*x*^2^ = 2.42; df = 1; *P* = 0.119) and FD environments respectively (*x*^2^ = 0.402; df = 1; *P* = 0.525). Exposure to α-pinene had rather minor effect on both the oviposition (*x*^2^ = 0.019 and *x*^2^ = 0.505; df = 1; *P* = 0.889 and *P* = 0.477) and post-oviposition (*x*^2^ = 2.274 and *x*^2^ = 0.834; df = 1; *P* = 0.132 and *P* = 0.361) period of females experiencing FD and DR conditions respectively (Supplementary Figure 2).

## Discussion

Our results demonstrate that (a) exposure to the aroma of the plant metabolite α-pinene affects major life history traits such as lifespan and reproduction in adult olive flies, (b) the effects of α-pinene are modulated by both the gender of adults and the dietary context. Exposure to α-pinene (c) increases longevity only on dietary restricted males but has no apparent effect on female longevity. Interestingly, the aroma of α-pinene (d) increases fecundity of dietary restricted females only and modulates age–specific patterns of egg laying on females fed in full diet conditions. Therefore, our hypothesis that exposure to α-pinene increases longevity and reproduction of adult olive flies regardless of gender and dietary context should be rejected.

### A-pinene modulates life-history traits and chemosensory stimulation

To our knowledge this is the first report demonstrating that a plant compound affects key life history traits of adult olive flies, such as longevity, lifetime and age-specific reproduction, through olfaction. Our experimental manipulations were designed to minimize any direct contact of α-pinene with the gustatory system of the exposed individuals. Although, we cannot exclude that in FD treatments, food odors could have an effect and overlay with effects of the odor of α-pinene, yet comparisons were performed within the same nutritional environments depicting a clear effect of α-pinene in extending longevity of males and increasing fecundity of DR females. The clear and more pronounced effect of the induced phenomenon in adults fed only sugar (free of any odors) further strengthens our conclusions. Therefore, we strongly suggest that the observed effects result from the stimulation of *B. oleae* chemosensory system that is triggered by the detection and olfaction of α-pinene aroma. Apparently future studies should concentrate on exploiting the underlying physiological and/or molecular mechanisms of the above phenomenon.

As it is stated earlier, the aroma of α-pinene is known to elicit behavioral changes in both male and female olive flies. Perception of α-pinene molecules by the olfactory system of *B. oleae* males could modulate the activity of neurotransmitters and of different peptide or steroid hormones^21^,^22^,^36^, which in turn may affect different homeostatic mechanisms that regulate longevity, as shown in other model organisms^37^,^38^. Compelling evidence that chemosensory systems (mainly olfactory but also gustatory) can modulate longevity has been reported in both *C. elegans* and *D. melanogaster*^20^,^24^,^26^,^39^,^40^.

For females exposure to the aroma of α-pinene modulates lifetime fecundity of DR females and generates differences in age-specific egg-laying patterns of FD ones. Interestingly our study demonstrates differential results of females held in rich and poor food conditions. Although exposure of females to α-pinene ended on day 16^th^ of life, effects on patterns of egg-laying were evident weeks or months later. Previous studies report stimulatory effects of α-pinene on initiation of oviposition^30^ and induction of electrophysiological responses on females^31^. The odor of various plant volatiles can act either as an ovipositional stimuli and attractant or as a repellent^18^, (reviewed in^41^). Although evidences regarding the underlying regulative mechanisms do not exist, it is plausible to argue that the aroma of α-pinene may serve as an olfactory input, which activates transcription factors (e.g., DAF-16/FOXO, insulin, steroid signaling) and triggers release of secondary messengers (e.g., hormones) that regulate physiological changes in female olive flies, some of which modulate longevity and reproductive functions^23^,^42^. Direct contact (feeding or application) of plant compounds (e.g plant adaptogens, phytochemicals) can have profound effects on life prolonging and shaping life-history traits (larval development, fecundity) by activating common signaling pathways^9^ with those activated through chemosensory stimulation (for reviews see)^23^,^36^,^38^. Further studies on the causal mechanisms underlying exposure to the aroma of α-pinene could provide useful information towards this direction.

### Diet-specific effects

Our results demonstrate that the beneficial effects of exposure to α-pinene depend on the dietary context of adult olive flies. Interestingly, our work goes in the opposite direction of the seminal study of Libert and co-workers^20^ where DR individuals that could smell food lost lifespan extension. In this work yeast odor (which is accepted as an exclusively nutritional cue for many organisms including insects) has been used while in our work a species-specific odorant stimulus (α-pinene) is involved, which elicits various behavioral and physiological responses (see introduction) but is not connected with adult feeding. A-pinene may activate ‘private’ mechanisms (i.e. organism-specific) of lifespan extension and not ‘public’ ones, whose effects are still unknown for olive fly^43^. Private mechanisms influencing lifespan refer to unique links developed between specific chemical stimuli (usually closely related to ecological challenges each species faces) and receptors, while ‘public’ ones (evolutionary conserved across many taxa) refer to links that arise due to response of organisms to fundamental needs, such as the need for food and mates. Furthermore, in the study of Libert and co-workers DR individuals had access to low levels of yeast (protein), whereas in our study DR individuals were deprived of yeast. Even small amounts of yeast may evoke the expression of different genes, which could account for the opposite effects observed in our study. Diet composition has a significant impact on lifespan and reproduction and may interact with other factors. Similar to other studies on tephritids^44^ (and references therein) absence of yeast hydrolyzate in adult diet decreases lifespan by 18.41% in male olive flies (FD vs DR). Longevity extension was observed for males exposed to the aroma of α-pinene, though differences were significant only in DR males. Variation in longevity extension observed between DR and FD exposed males may be attributed to trade-offs between behavioral and physiological processes related to adult food.

For females direct prolongevity effects after exposure to α-pinene have not been depicted. Although, reproduction does not predict lifespan and these two can be uncoupled^45^,^46^ (and references therein) trade-offs between them may still exist to some extent^47^,^48^,^49^. In many studies artifactual lifespan enhancement has been observed as a result of reduced fecundity. In our study, we found an increase in lifetime fecundity of DR females, without longevity trade-off. This suggests that α-pinene could promote longevity.

On the other hand, yeast availability in female food diet could mask measurable/quantitative trade-offs between reproduction and longevity. In our study, FD olive fruit fly females laid significantly more eggs and lived longer than DR ones, despite the increased reproduction cost, demonstrating the high impact of full diet on female fitness. Yeast by itself is a strong mediator of lifespan and fecundity in female fruit flies^50^,^51^. Full diet conditions may drive fecundity and longevity extension to a plateau that exposure to the aroma of α-pinene could not enhance further. As a consequence beneficial effect of α-pinene aroma could be difficult to detect and could be represented as changes in other life history traits. A shift of age-specific fecundity pattern towards early reproduction of females exposed to α-pinene aroma may serve as a support to the above arguments. Age specific patterns of egg laying in olive fly and other fruit flies are quite plastic and subject to several external and internal factors. We have recently shown that female fecundity is greatly regulated by seminal fluid and the mating history of male patterns and that effect may be long lasting^52^.

### Sex-specific effects

Another interesting outcome of our study was the differential response of the two sexes to the aroma of α-pinene. Life-prolonging effects have been shown for males but not for females where reproductive traits such as fecundity have been regulated. Since males were deprived of females, they were prevented of costly reproductive activities such as courting and mating. Therefore, beneficial stimulatory effects of exposure to α-pinene may have been directed to somatic maintenance and longevity extension. On the other hand, since females are capable of producing and laying eggs without the involvement of males, they had to balance the “allocation” of beneficial effects of the aroma of α-pinene between reproduction and lifespan, as discussed.

Differential responsiveness of males and females could also possibly have arisen because of activation of different mechanisms and/or signaling pathways. A-pinene aroma through the stimulation of the neurosensory system of *B. oleae*, could lead to secretion of same or different kinds of hormones in males and females, whose effects may regulate different physiological processes (homeostasis, reproduction, feeding etc.). Additionally, different activated hormonal pathways could act both separately or coordinately to influence both longevity and reproduction. For example juvenile hormone regulates multiple aspects of physiology (and behavior), representing remarkably variable functions between males and females of *Drosophila melanogaster* (for review see^53^). Other possible explanations on differential response between genders to olfaction cues sensing (e.g. CO_2_ sensing see^24^) include differences between the two sexes in the olfactory stimulation/sensitivity and/or in the procession of odor information.

### A-pinene may regulate ecological responses of adult olive flies

A-pinene emitted from olive fruit may represent a cue informing *B. oleae* females about fruit abundance and suitability and, therefore, favorable conditions for larvae survival. A-pinene aroma acting synergistically with female pheromone attracts males^29^ and increases the mating success on both male and female olive flies^32^. Once females mated, α-pinene stimulates and increases fecundity or modulates age-specific patterns of egg laying, boosting early reproduction and maximizing individual fitness. On the other hand, increased longevity observed in males, coupled with higher mating success^32^, may contribute to a higher number of copulations during lifetime; either for replenishment of the sperm reservoirs of already mated females or fertilization of virgin females. Differential effects of α-pinene aroma in the two dietary contexts, could reveal plastic responses generating alternate life history strategies, which ultimately enhance the chances of survival and reproduction under available resources^37^ or serve as sensory cue triggering mechanisms towards cell protection, allowing animals to react quickly in rapid changing environmental conditions.

## Materials and Methods

The experiments were conducted during autumn 2013 in the laboratory of Entomology and Agricultural Zoology at the University of Thessaly, Greece at 25 ± 1 °C, 65 ± 5% R.H. and a photoperiod of L14:D10. Light was provided by daylight fluorescent tubes and by natural light from four windows with the intensity inside the test cages ranging from 1500 to 2000 Lux. All flies used were obtained from infested fruit (collected in the area of Volos, Greece) and were reared in the above standard laboratory conditions on olive fruit (female oviposition and immature development) for one generation (*F*_1_). Using an aspirator, adults were separated by sex within one-day post emergence that is several days before attaining sexual maturation^32^. Experimental flies experienced a simulated dawn and dusk during which the light intensity ramped up and down during 1 h period, respectively. We used unmated flies (males and females) to avoid potential effects of mating on longevity and egg production that could confound our results. Especially for female tephritids, male insemination is not necessary for egg production^52^,^54^,^55^ and virgin females are widely used^35^,^44^,^56^ to avoid possibly confounding components costs, such as potential mortality risk due to copulation, exposure to males and thus to chemical communication between them, that are associated with copulation in the olive fly and other female insects^52^,^57^,^58^.

### Effect of α-pinene and protein availability on male longevity

Soon after sexing males were placed in groups of 40 in plexiglass cages (20 by 20 by 20 cm bearing two 225 cm^2^ windows covered with mesh cloth for ventilation) with water and adult food (ad libitum) consisting of either a mixture of yeast hydrolyzate (MP Biomedicals LLC, France), sugar and water at 1:4:5 (FD conditions) or a mixture of sugar and water (DR conditions). On day 14 of adult life, 10 adults of each food regime were transferred into small cubic plexiglass cages (15 by 15 by 15 cm bearing a round opening of 7.5 cm in diameter on one side that served as the entrance to the cage) and exposed to 20 μl (-)-*α*-pinene (98% purity, Alfa, Aesar, France) or water for control, for three successive days. Comprehensive details and exact procedure of exposure to α-pinene are given by Gerofotis^32^. Therefore, we established four male cohorts that experienced FD conditions and were either exposed (a) or non-exposed (control) to α-pinene (b) and DR conditions and were either exposed (c) or non-exposed (control) to α-pinene (d). On day 17 (one day after last exposure to α-pinene), males of each group were transferred and held individually till the end of adult life into transparent plastic cages (400-ml capacity plastic cups) bearing a 24 cm^2^ lateral opening covered with mesh cloth to allow adequate ventilation and offered the respective adult food they had experienced before. Male lifespan was recorded daily until the death of last individual in the cohort. Fifty individuals were considered for each treatment.

### Effect of α-pinene and protein availability on female fitness

Following the procedures described above we established four female cohorts: females that experienced FD conditions and were either exposed (a) or non-exposed (control) to α-pinene (b) and DR conditions that were exposed (c) or non-exposed to α-pinene (d). On day 17 (one day after last exposure to α-pinene), females of each group were transferred and held individually into transparent plastic cages (similar to those described above) maintaining them with the respective adult food they had experienced till then. As oviposition substrates we used five 18 mm, yellow, hollow, ceresin hemispheres (domes) that were fitted into five holes of respective size perforated on the base of each female cage (see^52^). Egg production and female age at death were recorded until the death of last individual in the cohort. Fifty individuals were considered for each treatment.

### Data analysis

Kaplan-Meier estimators followed by the log-rank test were used for modeling and comparing lifespans of both males and females, and oviposition and post oviposition periods of females. Pairwise log-rank tests were adjusted for multiple comparisons. Poisson regression was used for the assessment of the effect of α-pinene and protein availability on fecundity. Squared Wald test results for the model parameters are presented. The comparison of oviposition distributions in a pairwise fashion, between exposed and non-exposed individuals within each diet regime, was assessed through ROC (Receiver Operation Curve) analysis along the lines presented in Alonso^59^. Chi-square was used to compare the proportion of ovipositing females in a pairwise manner among the four female cohorts. For all analyses, p-values less than 0.05 were considered statistically significant. SPSS 21.0 (IBM Corp., Armonk, NY) and R 3.1.3 (R Core Team, Vienna, Austria) were used for data analysis.

## Additional Information

**How to cite this article**: Gerofotis, C. D. *et al*. The odor of a plant metabolite affects life history traits in dietary restricted adult olive flies. *Sci. Rep.* **6**, 28540; doi: 10.1038/srep28540 (2016).

## Supplementary Material

Supplementary Information

## Figures and Tables

**Table 1:** Longevity parameters of adult olive flies that were exposed and non-exposed to the aroma of α-pinene and held in diet restriction (DR) and full diet (FD) food conditions.

Adult cohort (Number of individuals)	Longevity parameters in days ± SE			
	Average	Quartiles		
		25	50	75
**Males**				
FD				
Exposed (n = 48)	56.37 ± 5.01a	58 ± 9.00	47 ± 2.15	36 ± 1.12
Non-exposed (n = 47)	51.42 ± 4.74a	54 ± 3.73	45 ± 3.42	33 ± 1.99
DR				
Exposed (n = 49)	49.63 ± 2.76a	61 ± 3.51	49 ± 4.49	37 ± 2.63
Non-exposed (n = 47)	41.95 ± 1.87b	50 ± 1.60	42 ± 2.28	33 ± 2.98
**Females**				
FD				
Exposed (n = 44)	78.92 ± 6.03a	114 ± 25.97	60 ± 2.94	48 ± 3.61
Non-exposed (n = 53)	83.96 ± 6.75a	132 ± 19.69	64 ± 5.09	46 ± 5.96
DR				
Exposed (n = 50)	59.68 ± 3.70a	66 ± 3.41	53 ± 3.03	46 ± 3.54
Non-exposed (n = 51)	58.87 ± 3.09a	64 ± 3.39	56 ± 2.28	46 ± 1.56

Within sex, numbers followed by different letters are significantly different (pairwise comparisons log-rank test, P < 0.05).
