# Supplementary material for: The odor of a plant metabolite affects life history traits in dietary restricted adult olive flies
Source: Sci Rep. 2016 Jun 24;6:28540. doi: 10.1038/srep28540 (PMC4919778; doi:10.1038/srep28540)
Supplement: Supplementary Information [file srep28540-s1.pdf]

Submitted to:

Scientific Reports

The odor of a plant metabolite affects life history traits in dietary restricted adult olive flies

Christos. D. Gerofotis,[1] Charalampos. S. Ioannou[1], Christos. T. Nakas[2,3], and Nikos. T. Papadopoulos[1*]

[1]Laboratory of Entomology and Agricultural Zoology, Department of Agriculture Crop Production and Rural Environment, University of Thessaly, Phytokou St., 38446 N. Ionia Magnisia, Greece

[2]Laboratory of Biometry, Department of Agriculture Crop Production and Rural Environment, University of Thessaly, Phytokou St., 38446 N. Ionia Magnisia, Greece

[3]Inselspital - Bern University Hospital, Center of Laboratory Medicine, University Institute of Clinical Chemistry, ZLM INO F, Freiburgstrasse, 3010 Bern, Switzerland

Send correspondence to:

Nikos Papadopoulos[*]

Department of Agriculture, Crop Production and Rural Environment

Fytokou St., 38446 N. Ionia, Magnisia

Tel: +302421093285

Email: nikopap@uth.gr

Supplementary Figure 1. Event history diagrams

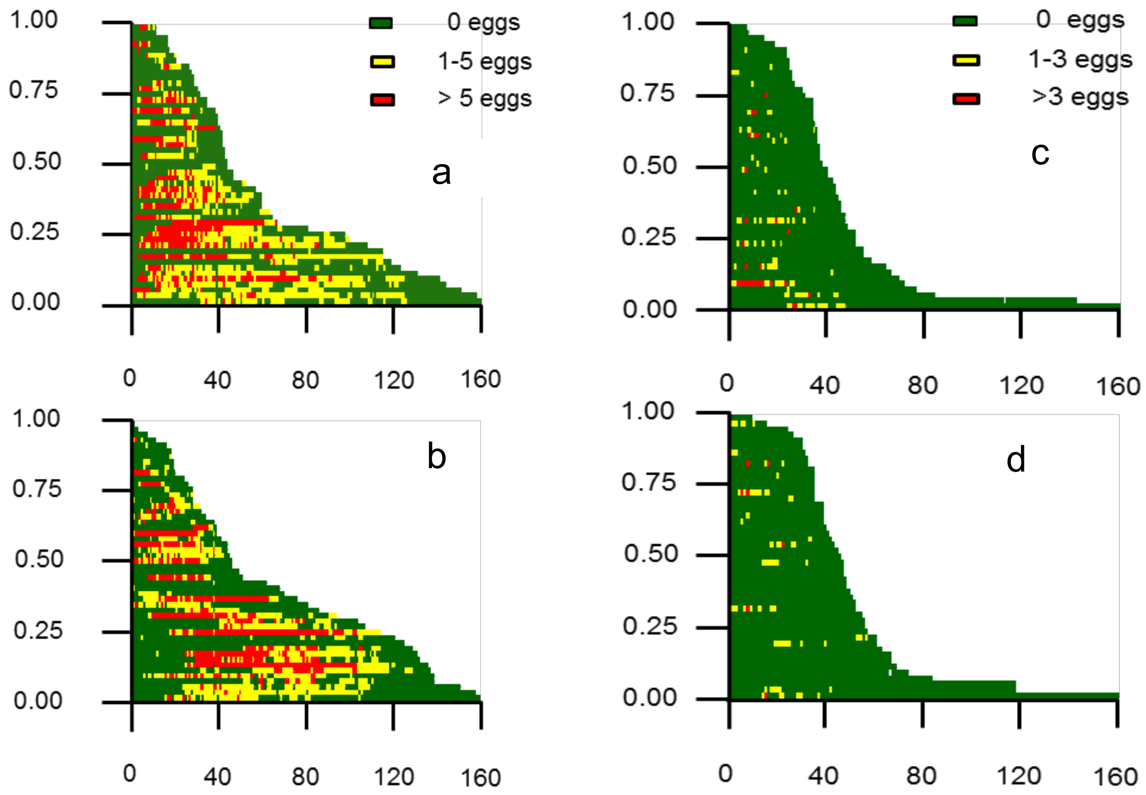

Supplementary Figure 2. Oviposition and post-oviposition periods

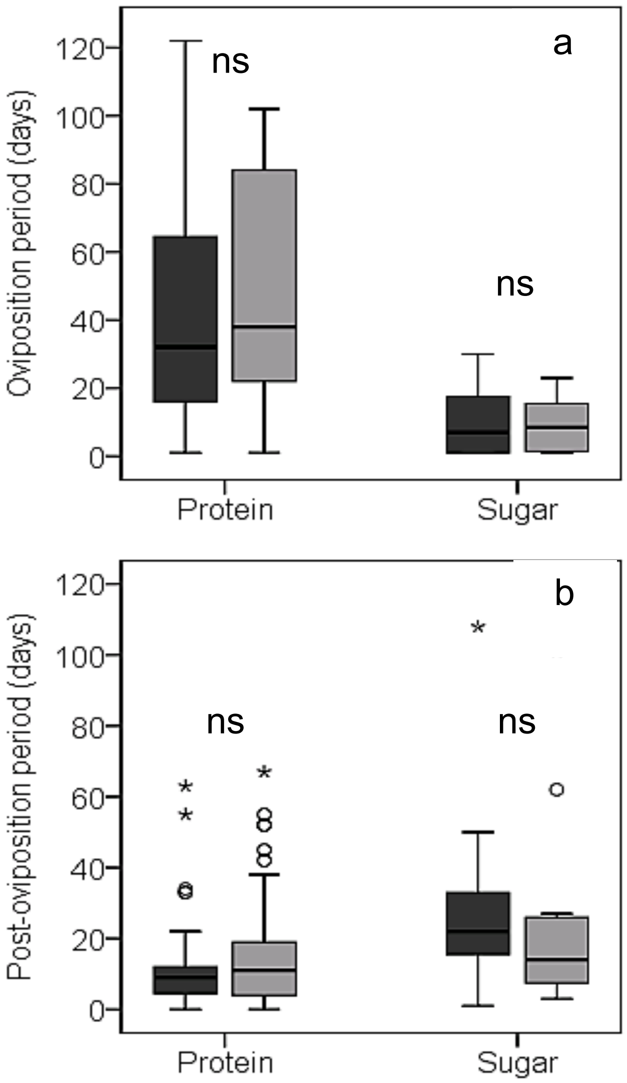

Supplementary Figure 3. Oviposition distribution

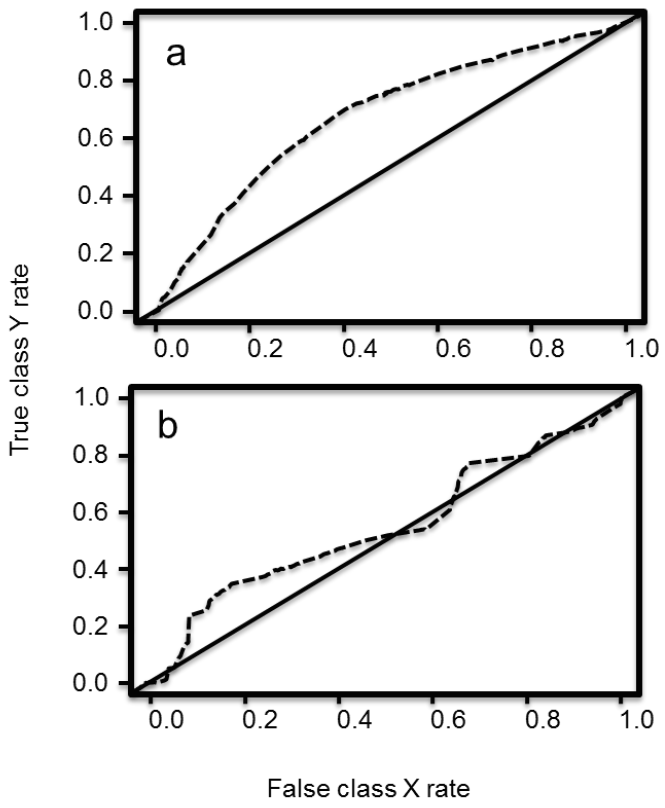

Figure 1S. Event history charts (Carey et al., 1998) for *Bactrocera oleae* females showing relationship of cohort survival and individual-level reproduction across treatments. Each horizontal line within a graph denotes a life line for an individual female, the length of which is proportional to her lifespan and with color-coded segments corresponding to age classes. The age-specific egg laying intensity for each individual female corresponds to the shading: green = zero eggs; yellow = 1–5eggs; red > 5 eggs for females fed in FD conditions (Fig a & b). Figure a represents exposed females to α-pinene whereas Fig b non-exposed ones. For females feeding in DR conditions, color scale has been modified and represent different laying intensities, green = zero eggs; yellow = 1–3 eggs; red >3 eggs for females fed in sugar (Fig c & d). Figure c represents exposed females to α-pinene whereas Fig d non-exposed ones.

Figure 2S. Box-plots representing oviposition (a) and post-oviposition (b) periods of females, exposed to the aroma of α-pinene (black boxes) and non-exposed (grey boxes) in the respective food regimes.

Figure 3S. Effect of exposure to α-pinene on age-specific fecundity. Receiver Operation Characteristics (ROC) analysis depicting the overlap of oviposition distribution between *Bactocera oleae* females a) fed in FD conditions-exposed vs. non-exposed to α-pinene (AUC = 0.67, *P* = 0.002) and b) fed in DR conditions- exposed vs non-exposed to α-pinene (AUC = 0.55, *P* =0.62)
